# Internal carotid artery rupture during endovascular thrombectomy in patients with tandem occlusion: A two-case report

**DOI:** 10.1177/19714009241252624

**Published:** 2024-05-10

**Authors:** Catarina Freitas Lopes, Lia Lucas Neto

**Affiliations:** Instituto de Anatomia, Faculdade de Medicina, 37811 Universidade de Lisboa. Lisboa, Portugal.

**Keywords:** Complicated endovascular thrombectomy, internal carotid artery rupture, subarachnoid haemorrhage, tandem occlusion

## Abstract

Endovascular thrombectomy in patients with tandem occlusions can rarely result in the rupture of the internal carotid artery, leading to subarachnoid haemorrhage and death. However, this complication and its causes are rarely reported and discussed in the literature. We describe two cases of internal carotid artery rupture during endovascular thrombectomy in patients with tandem occlusion. It is hypothesised that the primary approach to the distal lesion, before recanalization, creates a blind alley that faces an intraluminal pressure increase upon manual contrast injection, surpassing the vessel’s resistance and resulting in arterial wall rupture. To prevent this complication, approaches such as treating the proximal occlusion first, injecting the contrast through a microcatheter or retracting the endovascular support catheter proximally to the stenosis of the cervical internal carotid artery have been suggested and are discussed.

## Introduction

Endovascular mechanical thrombectomy has become the standard of care for patients with acute ischemic stroke due to large vessel occlusion. For tandem occlusions, simultaneously approaching extracranial and intracranial lesions has been shown to improve clinical outcomes and reduce mortality rates.^1^ It is generally considered advantageous to initially open the distal occlusion for rapid cerebral reperfusion, followed by treating the proximal extracranial occlusion.^1^ During this procedure, catheterization of the internal carotid artery and contrast injection are performed to confirm the catheter’s correct positioning and visualise the distal occlusion. However, in tandem occlusions, there is a risk of internal carotid artery rupture in its intradural portion when contrast is injected under pressure inside a closed system.^2,3^ This rupture leads to subarachnoid haemorrhage that frequently results in poor functional outcome or death. The published literature concerning this complication worldwide is very scarce and usually limited to congress presentations and single case reports.

## Case report

**Figure 1.**
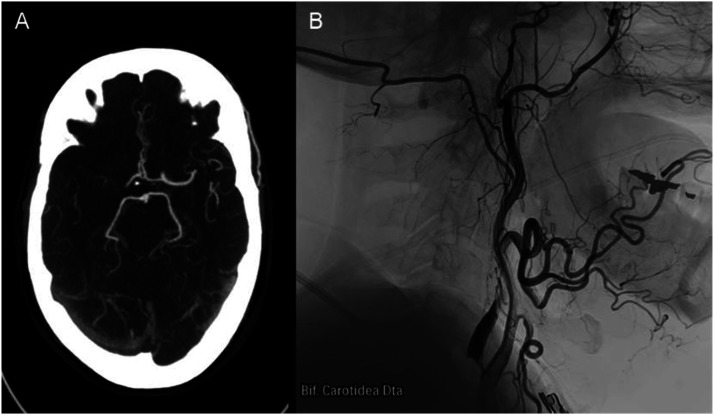
(A) Axial CT angiography depicts M1 occlusion of the right middle cerebral artery and distal segment of right internal carotid artery. (B) Lateral view cervical angiography reveals right internal carotid artery atheroma plaque and post bulbar occlusion.

Two patients, a 63-year-old male and an 84-year-old female, presented with symptoms and clinical signs suggestive of middle cerebral artery stroke, with NIHSS score of 14 and 24, respectively. Both patients exhibited a history of arterial hypertension, along with distinct occurrences of esophageal and colon cancer. Angiotomography confirmed ischemic stroke with M1 middle cerebral artery and internal carotid artery terminal segment occlusion, along with post-bulbar complete occlusion of the ipsilateral internal carotid artery (Figure 1). As they did not meet the criteria for administering recombinant tissue plasminogen activator, isolated endovascular thrombectomy was performed.

**Figure 2.**
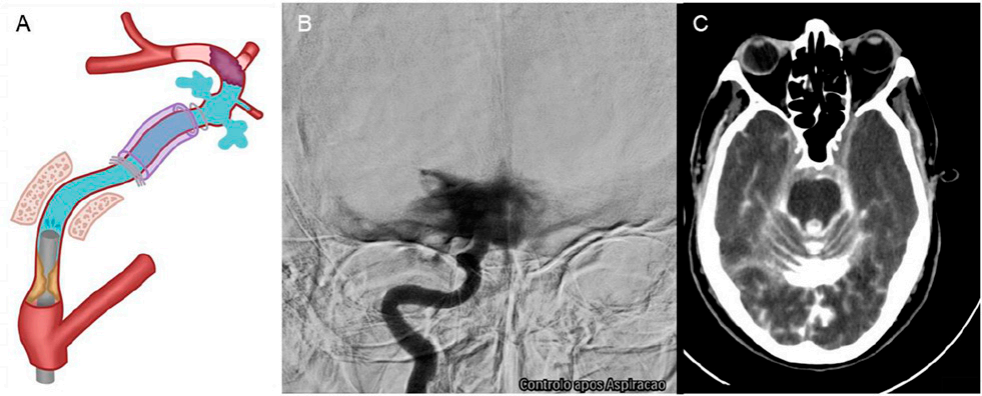
(A) Graphic representation of the internal carotid artery’s wall rupture beyond the distal dural ring. (B) Extensive contrast extravasation and rupture of the internal carotid artery. (C) CT scan with extensive subarachnoid haemorrhage.

Triaxial catheterization of the internal carotid artery was carried out using a 6Fr 088 guiding catheter and a 6Fr over a 3.8Fr aspiration catheter and J shaped .014″ guidewire. In both cases, following the first aspiration attempt without any clot retrieval, the manual injection angiographic control revealed extensive contrast extravasation and radiological signs of internal carotid artery wall rupture (Figure 2).

One case underwent arterial occlusion using six coils to stop the active bleeding, while the second was managed with temporary reduction of blood pressure and palliative measures. The procedures were stopped, and confirmation of this complication was immediately obtained through a flat panel cone beam CT scan on the angiography table, followed by a CT scan, which showed extensive subarachnoid and intraventricular haemorrhage (Figure 2).

Neither case warranted a neurosurgical approach. Both patients experienced hemodynamic instability, worsening clinical status, increased NIHS scores, severe brain dysfunction, and died a day after the endovascular thrombectomy complication.

## Discussion

Most documented cases of intracerebral haemorrhage during endovascular thrombectomy are usually distal and result from arterial wall perforation by a microwire, microcatheter or stent retriever.^4^ In our reported patients, after attempted aspiration thrombectomy, the internal carotid artery ruptured in its post-cavernous segment, during regular manual contrast injection for angiographic control. The catheters did not pass the thrombus and, additionally, the guidewire was J-shaped and went smoothly through all the procedure. Nevertheless, in both cases, the absence of clot aspiration during the initial attempt entails a period, during injection, in which a distal and proximal internal carotid occlusion occurred simultaneously.

Upon entering the intracranial region, the internal carotid artery loses its external elastic lamina at the cavernous segment (C4) and acquires a thinner adventitia.^2^ This thinner arterial wall, after the distal dural ring, may render it more susceptible to rupture, especially in the presence of increased intraluminal pressure or arterial wall injury.^3^ Structural changes such as aneurysms can contribute to this type of complication.^5^ Yet, in these cases, the rupture is believed to be caused by contrast injection, which fills the blind alley/closed system formed due to tandem occlusion, leading to a significant increase in intraluminal pressure that exceeds the arterial wall resistance.^3,5^

To prevent this complication, one option is to initially treat the proximal lesion, with angioplasty and/or stenting, although this approach delays recanalization of the intracerebral distal blood flow. However, if the guiding catheter can traverse the occlusion site, as observed in our cases, a retrograde approach may be deemed preferable. Nonetheless, it is imperative to acknowledge that this approach creates a blind alley after the guiding catheter insertion in the internal carotid artery, with the vascular circuit occluded by the distal lesion and proximal carotid stenosis plus the diameter of the guiding catheter.^3,5^ Another option would be a low flow manual injection or the injection through a microcatheter, to avoid excessive increase in intraluminal pressure during contrast injection.^5^ Additionally, the guiding catheter may be retracted proximally to the stenosis of the internal carotid artery, while injecting contrast, preventing the formation of a blind alley but potentially resulting in a lower quality angiographic results.

## Conclusion

Endovascular thrombectomy is an effective treatment for most tandem occlusions, allowing for early reperfusion of distal territories. However, in rare cases, the procedure can create a closed arterial system that leads to intradural internal carotid artery rupture upon contrast injection due to a significant increase in intraluminal pressure, resulting in fatal subarachnoid haemorrhage. To ensure the safety of this procedure, further studies are needed to compare the retrograde approach (initially treating the distal lesion) with other endovascular thrombectomy techniques, to determine the approach with the lowest risk of internal carotid artery rupture. Additionally, a comprehensive analysis of the few registered cases could help identify other risk factors for this complication, enabling the implementation of preventive measures in high-risk cases.
